# Chloroplast RNA-Binding Protein RBD1 Promotes Chilling Tolerance through 23S rRNA Processing in Arabidopsis

**DOI:** 10.1371/journal.pgen.1006027

**Published:** 2016-05-03

**Authors:** Shuai Wang, Ge Bai, Shu Wang, Leiyun Yang, Fen Yang, Yi Wang, Jian-Kang Zhu, Jian Hua

**Affiliations:** 1 College of Plant Science, Jilin University, Changchun, P.R. China; 2 School of Integrated Plant Science, Plant Biology Section, Cornell University, Ithaca, New York, United States of America; 3 Tobacco Breeding and Biotechnology Research Center, Yunnan Academy of Tobacco Agricultural Sciences, Kunming, P.R. China; 4 Shanghai Center for Plant Stress Biology, Shanghai Institute of Biological Sciences, Chinese Academy of Sciences, Shanghai, P.R. China; 5 Department of Horticulture and Landscape Architecture, Purdue University, West Lafayette, Indiana, United States of America; 6 State key laboratory of Crops Genetics and Germplasm Enhancement, College of Agriculture, Nanjing Agricultural University, Nanjing, China; Peking University, CHINA

## Abstract

Plants have varying abilities to tolerate chilling (low but not freezing temperatures), and it is largely unknown how plants such as *Arabidopsis thaliana* achieve chilling tolerance. Here, we describe a genome-wide screen for genes important for chilling tolerance by their putative knockout mutants in *Arabidopsis thaliana*. Out of 11,000 T-DNA insertion mutant lines representing half of the genome, 54 lines associated with disruption of 49 genes had a drastic chilling sensitive phenotype. Sixteen of these genes encode proteins with chloroplast localization, suggesting a critical role of chloroplast function in chilling tolerance. Study of one of these proteins RBD1 with an RNA binding domain further reveals the importance of chloroplast translation in chilling tolerance. RBD1 is expressed in the green tissues and is localized in the chloroplast nucleoid. It binds directly to 23S rRNA and the binding is stronger under chilling than at normal growth temperatures. The *rbd1* mutants are defective in generating mature 23S rRNAs and deficient in chloroplast protein synthesis especially under chilling conditions. Together, our study identifies RBD1 as a regulator of 23S rRNA processing and reveals the importance of chloroplast function especially protein translation in chilling tolerance.

## Author Summary

Compared to cold acclimation (enhancement of freezing tolerance by a prior exposure to low non-freezing temperature), the tolerance mechanism to non-freezing chilling temperatures is not well understood. Here, we performed a genome-wide mutant screen for chilling sensitive phenotype and identified 49 candidate genes important for chilling tolerance in Arabidopsis. Among the proteins encoded by these 49 genes, 16 are annotated as having chloroplast localization, suggesting a critical role of chloroplast function in chilling tolerance. We further studied RBD1, one of the four RNA-binding proteins localized to chloroplast. RBD1 is only expressed in the green photosynthetic tissues and is localized to nucleoid of chloroplasts. Furthermore, RBD1 is found to be a regulator of 23S rRNA processing likely through direct binding to the precursor of 23S rRNA in a temperature dependent manner. Our study thus reveals the importance of chloroplast function especially protein translation in chilling tolerance at genome-wide scale and suggests an adaptive mechanism involving low temperature enhanced activities from proteins such as RBD1 in chilling tolerance.

## Introduction

Low temperature inhibits plant growth in general and limits the geographical distribution of plants. Earlier studies have identified numerous physiological and cellular changes associated with chilling (more than 0°C) or freezing (less than 0°C) conditions, such as alterations in membrane composition, calcium signals, metabolite composition, photosynthesis, and protective molecules [1,2]. Most of these changes are thought to help plants to cope with low temperature stresses. Plants differ in their abilities to tolerate chilling stresses. Low temperature often inhibits photosynthesis and reduces carbon uptake and allocation to developing sink tissues [3,4]. Many tropical and subtropical plants including maize, rice, and tomato do not survive at 4°C because they cannot undergo photosynthesis and carbon metabolism under this condition. Arabidopsis, as well as some overwinter cereals, can grow at the low temperature due to their biochemical and physiological adaptations which may include acclimation of photosynthetic metabolism [5,6].

Translation in chloroplast appears to be especially sensitive to chilling stresses. Chilling slows down protein biosynthesis in plastids by eliciting frequent ribosome pausing in tomato [7]. The otherwise chilling tolerant Arabidopsis plants become chilling sensitive when they are defective in chloroplast ribosomal biogenesis and RNA processing [8–12]. For instances, loss of the translation elongation factor SVR3, the rRNA maturation factor NUS1, and chloroplast RNA binding proteins CP29A and CP31A, all lead to increased chilling sensitivity through affecting chloroplast biogenesis [9,10,13]. In addition, the loss of chloroplast ribosomal subunits reduces the ability of plants to recover from prolonged chilling periods [11,12].

Chloroplast function is carried out by genes coded mostly by the chloroplast genome [14]. Transcription of such genes relies on two plastid RNA polymerases: nucleus-encoded RNA polymerase (NEP) and plastid-encoded RNA polymerase (PEP) [15,16]. Chloroplast RNAs need to be processed to become functional rRNAs and mRNAs. Many of the processing factors for RNA cleavage, splicing, editing or stability are RNA-binding proteins [13,17–19]. They are all coded by the nuclear genome. One family has pentatricopeptide repeats (PPR) and it usually carries out specific RNA processing in chloroplasts [19]. Another family contains RNA recognition motif/RNA-binding domain/ribonucleoprotein (RRM/RBD/RNP) domain and these proteins, referred to as RNPs, are suspected to regulate larger sets of RNAs [20]. Among the chloroplast RNPs, CP31A and CP29A are associated with a large pool of chloroplast transcripts and influence their stability, processing, and splicing [13].

While chilling tolerance mechanism is not well understood, cold acclimation, an enhancement of freezing tolerance by a prior exposure to low non-freezing temperature, has been intensively studied in *Arabidopsis thaliana* [21–25]. Cold acclimation is mediated in part by C-repeat binding factors (CBFs) which regulate the expression of a large number of *Cold Responsive* (*COR*) genes, some of which are thought to confer freezing tolerance. The upregulation of the *CBF* genes by low temperature is critical for this acclimation and is mainly modulated by *ICE1* (*Inducer of CBF expression 1*), *ICE2* and the three closely related *CAMTAs* (*Calmodulin binding Transcription Activators*) [26–29]. Although genes regulated by CBF proteins play vital roles in freezing tolerance, they only represent a small percentage of the *COR* genes. *CBF* independent regulations of cold acclimation or freezing tolerance have been found in Arabidopsis [30,31]. It is yet to be determined whether or not chilling tolerance and cold acclimation have shared mechanisms.

To have a better understanding of chilling tolerance in Arabidopsis, we carried out a chilling sensitive mutant screen on all available T-DNA insertion mutants that represent half of the total Arabidopsis genes. Interestingly, mutants defective in chloroplast localized proteins are overrepresented, indicating the importance of chloroplast function in chilling tolerance. Detailed characterization of one such mutant of a RNA binding protein supports a critical role of chloroplast translation in chilling tolerance.

## Results

### Screening for chilling sensitive mutants from a large collection of T-DNA mutants

**Fig 1:**
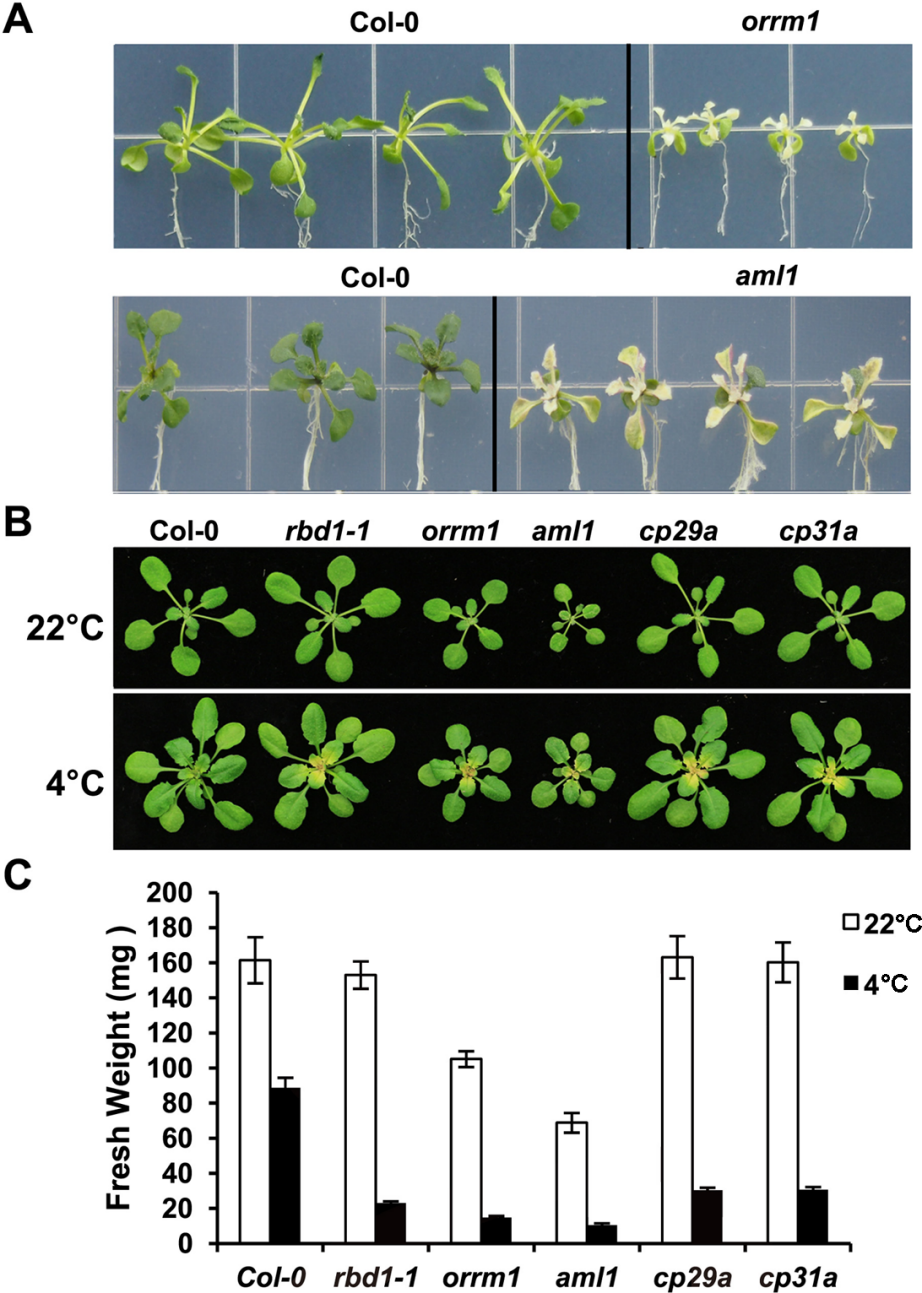
Chilling sensitivity of the *RNP* mutants. (A) Shown are wild type Col-0, *orrm1*, and *aml1* mutants grown for 2 months at 4°C on the plate under a 16 h light/day photoperiod. Representative chilling sensitive phenotypes are shown. (B) Shown are wild type Col-0, *rbd1-1*, *orrm1*, *aml1*, *cp29a*, and *cp31a* mutants grown at 22°C and 4°C. Top row: Plants grown for 3 weeks at 22°C under a 12 hour (h) light photoperiod. Bottom row: Plants grown for 3 weeks at 22°C, then shifted to 4°C for additional 4 weeks under a 12 h light photoperiod. (C) Shown are fresh weights of the 5 *RNP* mutants grown at 22°C and 4°C for 3 weeks and 10 weeks respectively under a 12 h light photoperiod.

In order to identify genes important for chilling tolerance in Arabidopsis at the genome scale, we analyzed 11,000 T-DNA insertion mutants covering half of the total Arabidopsis genes for chilling sensitive phenotypes. Each of them is putative homozygous T-DNA knockout or knockdown mutants in indexed genes generated by the SALK Institute [32] and available from the Arabidopsis Biological Resource Center (http://abrc.osu.edu/). The use of these indexed knockout or knockdown lines over chemical mutagenized population enabled the assessment and verification of phenotypes under different growth conditions and allowed uncovering of conditional lethal mutants and avoiding temperature-sensitive alleles of essential genes. In this screen (S1 Fig), four mutant seeds of each line were germinated and grown side by side with the wild-type Col-0 on vertical plates under a 16 hours (h) light/day photoperiod first at 22°C for 8 days and then transferred to 4°C for two months. Lines showing abnormal growth phenotypes compared to the wild type only at 4°C but not 22°C were selected as chilling sensitive mutants. These phenotypes include albino, yellow or purple leaves, abnormal leaf shapes, smaller leaves, and shorter roots. Because not all of these T-DNA lines were homozygous for the T-DNA insertion mutations, we further analyzed those lines where not all four seedlings exhibited mutant phenotypes to exclude those whose mutant phenotypes are not correlated with the T-DNA insertion. From this screen, a total of 54 lines showed growth defects at 4°C but not 22°C and we defined these mutants as chilling sensitive (Fig 1A).

We then examined the genes indexed to be disrupted by T-DNA insertions in these lines. Those with insertion in the promoter region but not exon, intron, or UTRs were removed as they might not affect the function of the indexed gene and the phenotype is unlikely due to a defect in the gene. This leaves us with 49 chilling sensitive mutant lines where the function or expression of the indexed genes are disrupted. None of the 49 genes are in the characterized CBF cold acclimation pathway, and loss of function mutants of *CBF1*, *CBF2*, *CBF3*, *ICE1*, *HOS1* and *SIZ1* were not in the collection. Although causal genes for chilling sensitivity may not be the indexed gene in a small proportion of the lines, we decide to analyze functional categories of these candidate genes as a group to reveal potential important chilling tolerance mechanisms. Among proteins encoded by the 49 candidate genes, 16 were annotated as localized to the chloroplast (Table 1), 10 to the nucleus, 6 to the cytosol, 4 to the mitochondria, and the rest either to other locations or without localization information. There is thus a high representation of chloroplast related genes in these mutants, suggesting a critical role of chloroplast function in chilling tolerance.

**Table 1:** Chilling sensitive mutants potentially defective in chloroplast-localized proteins.

T-DNA line	Gene ID	Insertion site	Notes
SALK_003066C	AT3G53460	exon	CP29; RNA-binding family protein
SALK_055951C	AT4G24770	exon	CP31; RNA-binding family protein
SALK_067017C	AT5G52440	300-UTR5	HCF106; Thylakoid membrane delta pH translocation pathway component protein
SALK_113136C	AT3G21300	exon	RNA methyltransferase family protein
SALK_071288C	AT1G05140	300-UTR5	Peptidase M50 family protein
SALK_090222C	AT4G10030	intron	ABHD11; alpha/beta-Hydrolases superfamily protein
SALK_107644C	AT2G37230	exon	Tetratricopeptide repeat (TPR)-like superfamily protein
SALK_018914C	AT3G22690	exon	YS1; TPR protein
SALK_072648C	AT3G20930	exon	ORRM1; a RIP and a RNA-binding family protein
SALK_074256C	AT5G23070	exon	ATTK1B; a thymidine kinase
SALK_075943C	AT5G52370	exon	Unknown protein
SALK_023468C	AT2G01918	exon	PQL3; required for NDH activity.
SALK_012657C	AT1G70200	exon	RNA-binding family protein
SALK_108952C	AT4G28210	300-UTR5	EMB1923; involved in embryo development
SALK_122911C	AT3G08920	exon	Rhodanese/Cell cycle control phosphatase family protein
SALK_013094C	AT4G31690	exon	Transcriptional factor B3 family protein

Among the 16 proteins with chloroplast localization, 4 were chloroplast RNP proteins and three were previously characterized: ORRM1 (AT3G20930), CP29A (AT3G53460) and CP31A (AT4G24770). Mutants of *CP29A* and *CP31A* were chilling sensitive and defective in processing various RNAs in chloroplast [13]. *ORMM1* is required for plastid RNA editing in Arabidopsis and maize [33], but its role in chilling tolerance has not been analyzed. The other RRM/RBD/RNP coding gene, AT1G70200, was not characterized before, and we named it *RBD1*. Mutants of all of these 4 genes showed bleaching in newly emerging leaves at 4°C. A mutant of another RNP coding gene *AML1* (AT5G61960) also had a bleaching phenotype. AML1 is predicted to localize in the nucleus but it may also have a chloroplast localization signal from the e-FP data. It plays a role in meiosis as well as in vegetative growth in *Arabidopsis thaliana* [34], but its role in chilling tolerance was not analyzed. We therefore further analyzed chilling sensitive phenotypes of mutants of these 5 RNP coding genes. When grown on soil at the normal temperature 22°C, the *rbd1*, *cp29a* and *cp31a* mutants exhibited a wild-type phenotype, while *orrm1* and *aml1* had a smaller size than the wild type Col-0 (Fig 1B). The *aml1* had yellow cotyledons at germination but recovered in a few days, and it often had white strips on the leaves. Consistent with visual phenotypes, the *rbd1*, *cp29a* and *cp31a* mutants has a fresh weight similar to that of the wild type at 22°C, but *orrm1* and *aml1* mutants displayed a 35% and 58% reduction compared to the wild type (Fig 1C). When shifted to 4°C after grown at 22°C for three weeks, new leaves emerged from all of these mutants were yellow (Fig 1B). When these mutants were grown constantly at 4°C in soil from germination, all their leaves were yellow and the plants were much smaller than the wild type with only 10% to 35% of the wild-type fresh weight (Fig 1C).

### *RBD1* is required for chloroplast function especially under chilling stress conditions

**Fig 2:**
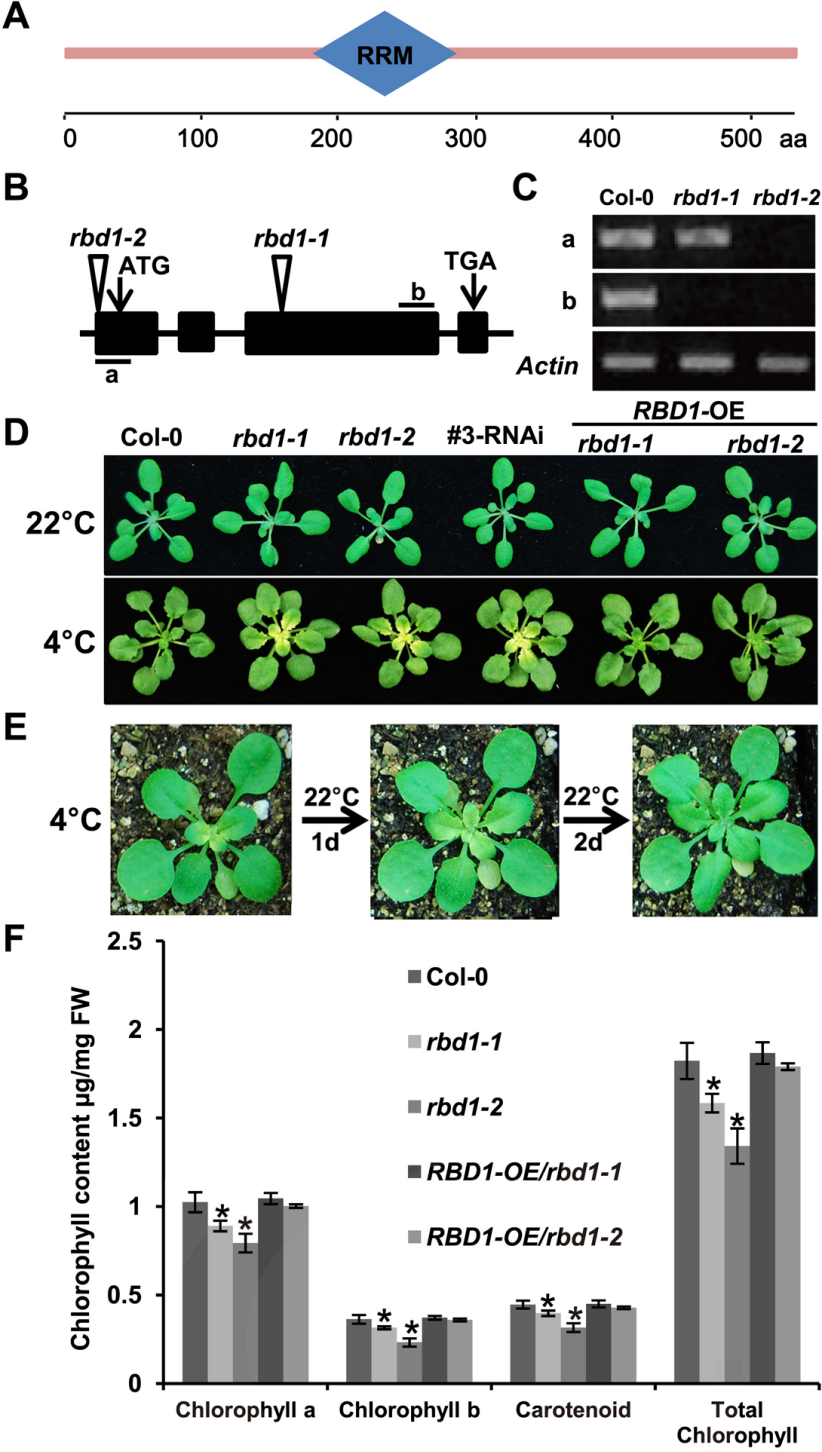
Characterization of the *rbd1* mutants. (A) Structure diagram of the RBD1 protein. It contains one RNA recognition motif/ RNA-binding domain between 188–267 amino acid (aa) predicted at SMART (http://smart.embl-heidelberg.de/). (B) Diagram of T-DNA insertion mutants of the *RBD1* gene. Arrows indicate the translation start (ATG) and stop (TAA) codons. Triangles indicate the site of T-DNA insertion in the SALK_041100 (*rbd1-1*) and SALK_012657 (*rbd1-2*) mutants. ‘a’ and ‘b’ mark the regions for RT-PCR analysis of *RBD1* gene expression. (C) Semi-quantitative RT-PCR analysis of *RBD1* expression in *rbd1* mutants. The *Actin* gene was used as a normalization control. Bands of each row are cut from the same gel. (D) Phenotype of *rbd1* mutants, *rbd1* RNAi and complementation lines. Top row: plants grown for 3 weeks at 22°C with 12h light /12h dark. Bottom row: plants grown for 3 weeks at 22°C, then shifted to 4°C for additional 4 weeks with 12h light /12h dark. (E) The *rbd1-1* plants at 2 days of 22°C recovery after 3 weeks of 4°C treatment. (F) The accumulation of pigments in *rbd1* mutants and complementation lines. Shown are contents of chlorophyll a, chlorophyll b and carotenoid from the fifth and sixth leaves of 2 weeks old plants grown at 22°C with constant light. Means ± SD, *n* = 3, * represents significant differences between means at P < 0.05 according to Student’s *t* test.

We chose *RBD1* for further analysis because there were no prior reports on this gene. The *RBD1* gene encodes a protein of 538 amino acids (aa) containing one RNA recognition motif/ RNA-binding domain (Fig 2A). We confirmed that the chilling sensitive phenotype observed in the mutant line SALK_041100 is due to the loss of the *RBD1* function through analysis of additional *RBD1* mutants and complementation test. The SALK_041100 line (named *rbd-1*) has the T-DNA inserted in the third exon of the *RBD1* gene, while the T-DNA insertion line SALK_012657 (named *rbd1-2*) has an insertion in the 5’-UTR of *RBD1* (Fig 2B). Both mutants were loss-of-function mutants of *RBD1* as no full length transcripts could be amplified by Reverse Transcription (RT)-PCR (Fig 2C). The *rbd1-2* mutant, like *rbd1-1*, produced yellow emerging leaves at 4°C (Fig 2D). In addition, RNA interference (RNAi) was used to reduce the expression of *RBD1*, and 13 of 15 RNAi transgenic lines produced yellow leaves under chilling conditions (Fig 2D). Furthermore, when the full-length *RBD1* cDNA driven by the cauliflower mosaic virus (CaMV) 35S promoter was transformed into the *rbd1* mutants, the chilling sensitive phenotype was suppressed in 30 of 32 the transgenic lines (Fig 2D). Therefore, the loss of *RBD1* function does confer chilling sensitivity.

We characterized the growth phenotypes of the *rbd1-1* mutants in more detail. When plants were shifted from three weeks’ growth at 22°C to 4°C, the yellowing or bleaching became visible only after 2 weeks of chilling treatment and only happened in leaves emerged at 4°C. There is a gradient of yellowing decreasing from the youngest leaf to the oldest leaves (Fig 2D). This yellowing phenotype is reversible, when plants were moved from 4°C back to 22°C, the pale green phenotype disappeared in 2 days (Fig 2E). The *rbd1-2* mutant also had a lighter green appearance compared to the wild type when grown constantly at 22°C. Using spectrophotometer, we found that the two *rbd1* mutants had a 10–20% reduction of chlorophyll a, chlorophyll b and carotenoids compared to the wild-type Col-0. This phenotype was not observed in the complemented lines (Fig 2F). Therefore, the *rbd1* mutants are compromised in chloroplast function especially under chilling conditions.

### Loss of *RBD1* does not affect the expression of *CBF* or *COR* genes

**Fig 3:**
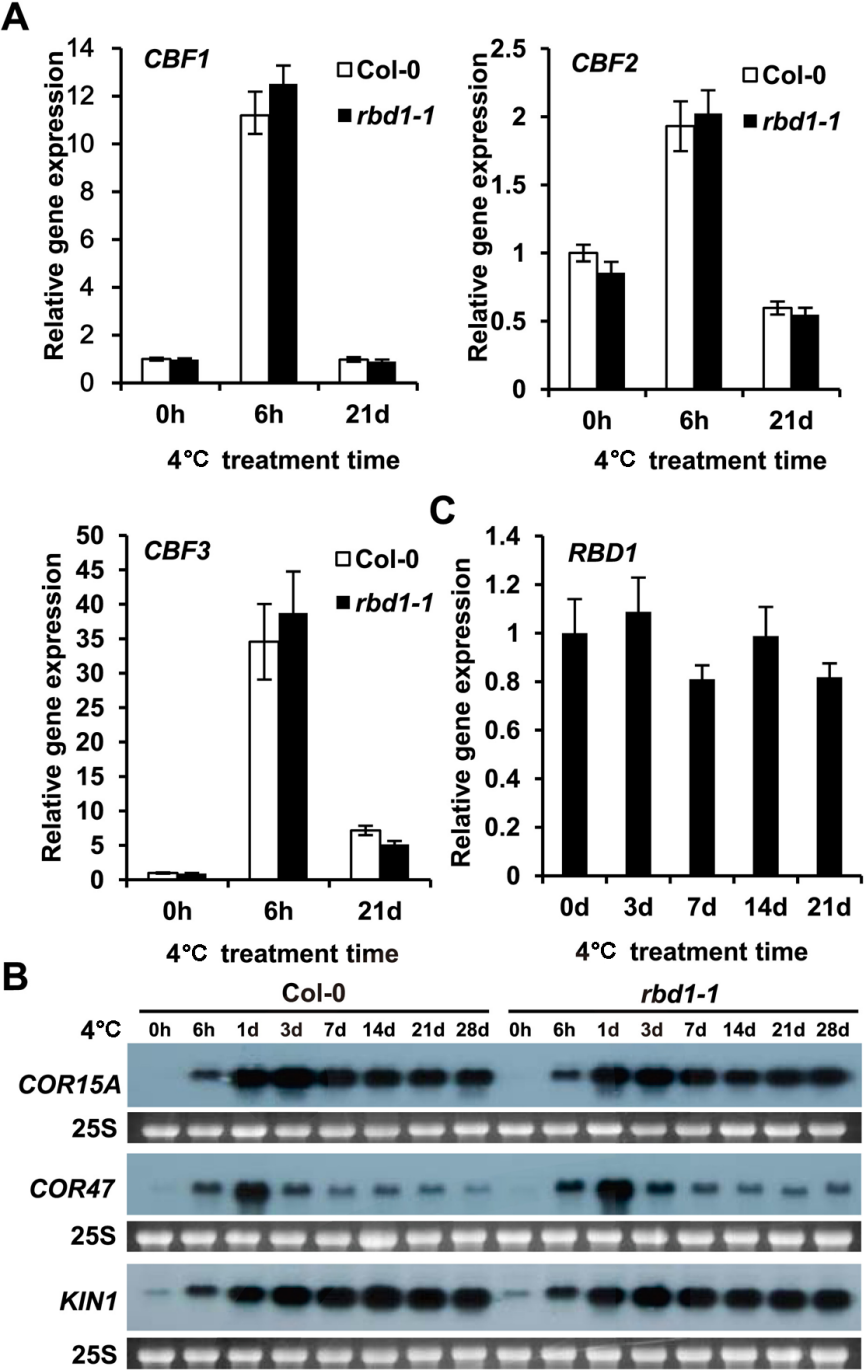
Expression of *CBF* and *COR* genes in *rbd1-1* mutant. (A-B) Expression level of *CBF1*, *CBF2* and *CBF3* (A) and *COR15A*, *KIN1* and *COR47* (B) after 4°C treatment at different time points. RNAs were isolated from five newly emerging leaves from 2 weeks old plants grown at normal condition followed by 4°C treatment. The *Actin* gene was used as a normalization control and the experiments were repeated three times with similar results (A). Equal loading was controlled by cytosolic 25S rRNA (25S) stained with ethidium bromide (B). (C) The expression patterns of *RBD1* under 4°C. Total RNA was extracted from two weeks old plants chilling-treated for 3, 7, 14 and 21 days. The *Actin* gene was used as a normalization control. The experiments were repeated three times with similar results.

Because the *rbd1* mutants displayed chilling sensitivity, we analyzed whether chilling induction of the *CBF* and *COR* genes was altered in the *rbd1* mutants. *CBF1*, *CBF2*, and *CBF3* are induced by 4°C treatment at 6 hours to the same extent in Col-0 and *rbd1-1* (Fig 3A). Similarly, these genes fall to the basal level after 3 weeks of 4°C treatment in both the wild type and the mutant (Fig 3A). *COR15A*, *COR47*, and *KIN1* genes are regulated by the *CBF* genes, and they were induced by 4°C treatment at 6 hours and stayed elevated during the subsequent chilling treatment for four weeks (Fig 3B). The *rbd1-1* mutant had the same induction kinetics and amplitudes of these three *COR* genes by 4°C treatment (Fig 3B). Therefore, the *rbd1-1* mutant is not impaired in its capacity to induce the *CBF* or *COR* expression by 4°C and the chilling sensitivity of the mutants is likely independent of the *CBF* pathway.

### The expression pattern of the *RBD1* gene

**Fig 4:**
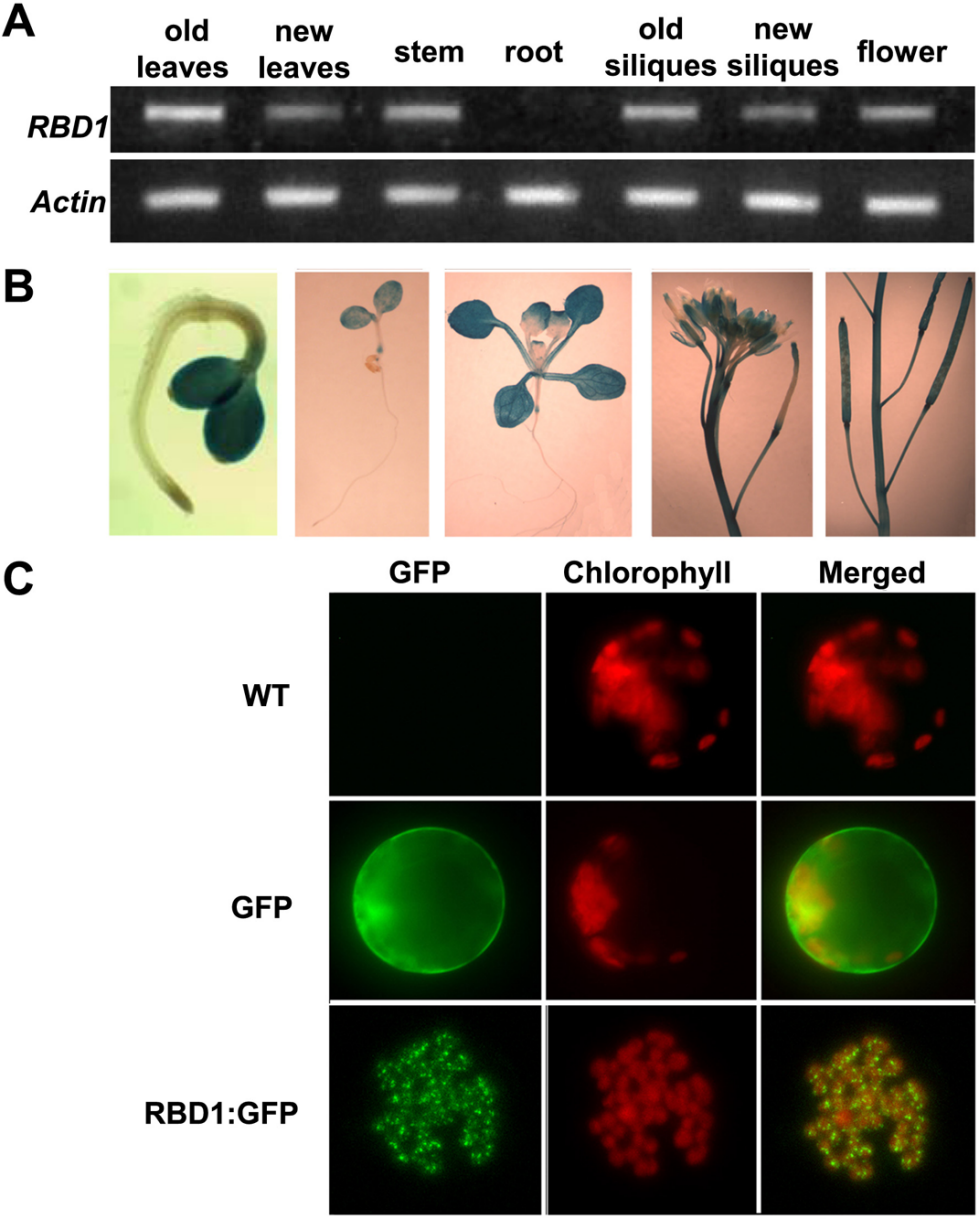
Gene expression and protein localization of RBD1. (A) Detection of *RBD1* transcripts in various tissues. Total RNA was extracted from the indicated tissues from plants grown at 22°C with constant light. The *Actin* gene was used as a normalization control. (B) GUS staining of pRBD1::GUS transgenic plants. (C) Shown are confocal microscope images of protoplasts isolated from wild-type Arabidopsis leaves either untransformed or transformed with GFP or RBD1:GFP. A representative image of a single protoplast is shown in each panel. Left panels show the GFP signals, middle panels show the chlorophyll signals, and the right panels show the merged signals.

The *RBD1* gene is expressed in green tissues. Using RT-PCR analysis, *RBD1* mRNA was detectable in leaves, stems, flowers and siliques but not in roots (Fig 4A). The expression pattern of *RBD1* was further investigated in transgenic lines containing a ß-glucuronidase (GUS) reporter gene fused to the *RBD1* promoter. GUS expression was observed in all green tissues throughout development (Fig 4B). In 3-day-old seedlings, GUS staining was primarily detected in emerging cotyledons (Fig 4B). In 14-day-old seedlings, it was detected in most of the plant but not in roots, and was particularly strong in cotyledons (Fig 4B). GUS staining was also observed in the stems and siliques, but not in the matured seeds (Fig 4B). Within the flower, it was strong in the sepals and carpels, but not in the stamens and petals (Fig 4B). In addition, older green tissues usually had higher expression levels than younger green tissues, suggesting that *RBD1* might contribute to the function of mature green tissues. Altogether, these expression patterns show that *RBD1* is highly expressed in the photosynthetic tissues, which was in agreement with its putative roles in chloroplast function.

*RBD1* is not induced by light according to the expression data from Arabidopsis eFP Browser (http://bbc.botany.utoronto.ca). It is not induced by chilling treatment either. RT-PCR revealed that *RBD1* is expressed at the same level during the chilling treatment from day 0 to day 21 (Fig 3C). The *RBD1* gene therefore might be constitutively expressed at the transcript level.

### RBD1 is localized to the chloroplast

RBD1 is annotated as a chloroplast targeted protein, which could explain its expression in green tissues. To verify the subcellular localization of the RBD1 protein, we expressed GFP (Green fluorescent Protein) fusion of RBD1 under the 35S promoter (RBD1:GFP) in protoplasts isolated from wild-type Arabidopsis leaves and monitored fluorescence by confocal microscopy. While the GFP protein alone was present in the cytoplasm and nucleus, the RBD1:GFP fusion protein was found exclusively in chloroplasts (Fig 4C). In addition, the RBD1:GFP fusion protein was dispersed as small fluorescent particles within chloroplast (Fig 4C), which is reminiscent of nucleoid localization [35]. This localization pattern does not appear to be temperature-dependent. In both 22°C and 4°C, RBD1:GFP is only present in the nucleoid structure in chloroplast (S2 Fig).

### The *rbd1* mutants are defective in processing of 23S rRNA

**Fig 5:**
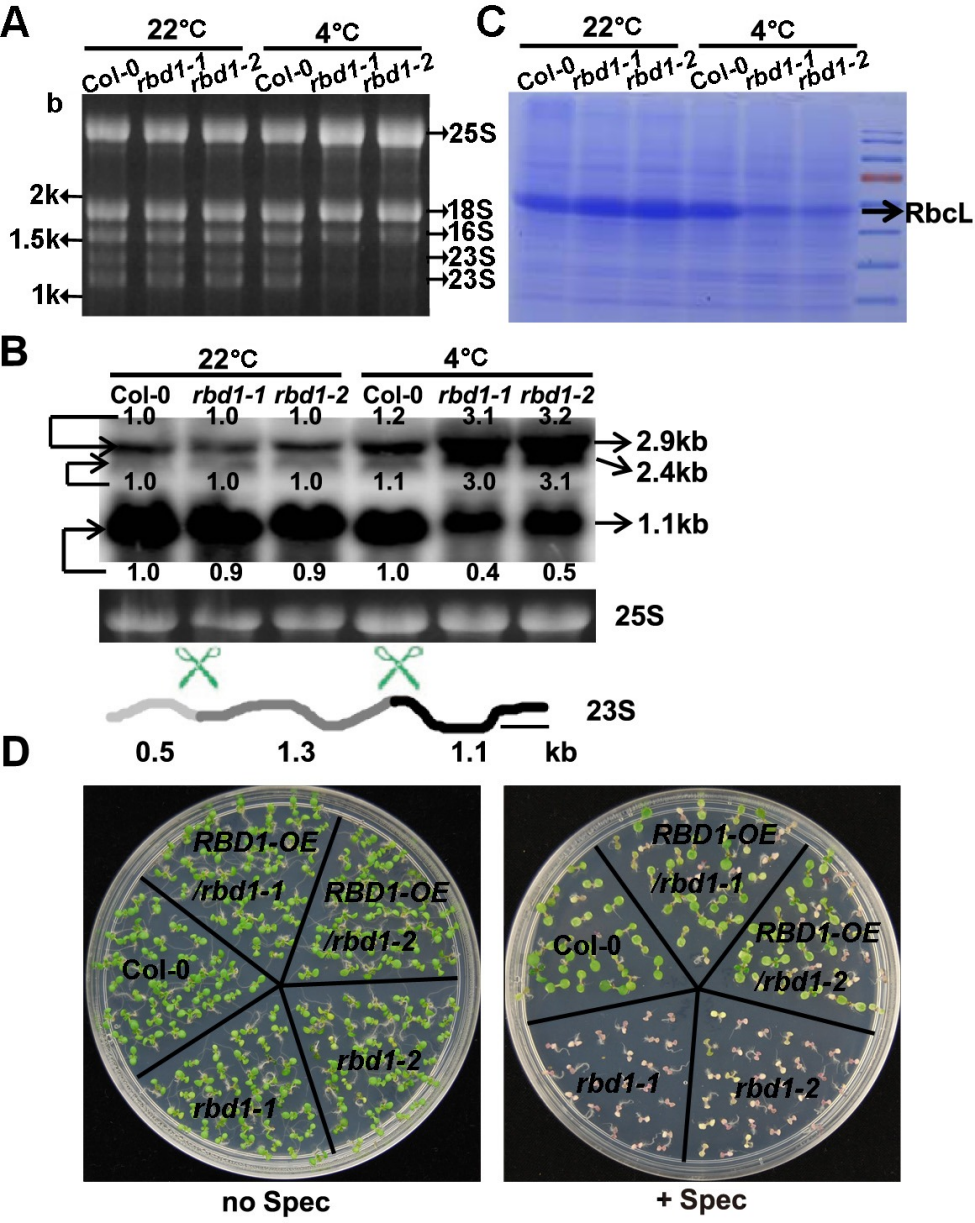
Chloroplast translation is inhibited in the *rbd1* mutants. (A) Total RNAs were fractionated on 1.2% formaldehyde gels and stained with ethidium bromide. (B) RNA blotting analysis of 23S rRNA processing. Total RNAs extracted from leaves were fractionated on 1.2% formaldehyde gels and hybridized to a probe (indicated by a black bar) detecting the 3’-end of the 23S rRNA. Equal loading was controlled by the cytosolic 25S rRNA (25S) stained with ethidium bromide. Processing sites in the 23S rRNA precursor were indicated by scissors. Marked numbers indicate the relative amount in the mutant compared to that in Col-0 quantified by Image J. (C) Coomassie Blue staining of total proteins from the wild type and the *rbd1* mutants after SDS-PAGE separation. Accumulation of Rubisco Large Subunit (RbcL) is indicated by an arrow. (D) Shown are Col-0, *rbd1* mutants and complementation lines grown on solid media in the presence or absence of spectinomycin at 3 mg/L for 7 days. For A, B, and C, plants were grown for 3 weeks at normal condition followed by 4°C for 4 weeks, and tissues were collected from five newly emerged leaves.

The nucleoid localization pattern and the yellowish leaf mutant phenotype promoted us to investigate the role of *RBD1* gene in chloroplast RNA processing because nucleoid is the site of rRNA processing and ribosome assembly [36]. We first analyzed total RNAs separated on formaldehyde denaturing agarose gel stained by ethidium bromide. Plastid rRNAs (23S, 16S, 5S, and 4.5S) and cytosolic rRNAs (25S, 18S, 5.8S, and 5S) can be easily observed on such gels because they are very abundant [37]. When the same amount of total RNAs was loaded on gel, *rbd1-1* and *rbd1-2* mutants showed significant reduced accumulation of the 1.1 and 1.3-kb species of the 23S rRNA compared to the wild type under chilling stress conditions, but exhibited almost the same amount of rRNAs as wild type at 22°C (Fig 5A).

RNA gel blot hybridization was used to further analyze the defects in 23S rRNA processing. The 23S rRNA with a full length of 2.9-kb is cleaved internally at two sites, yielding fragments of 0.5, 1.1, and 1.3-kb in wild type [38]. With a probe detecting the 3’-end fragments, we found that 4°C treatment did not affect the processing of 23S rRNA transcripts in the wild type (Fig 5B). In contrast, the chilling treated *rbd1* mutants accumulated the partially processed 2.9-kb and the 2.4-kb of 23S rRNA at a much higher level than the wild type, while the fully processed product of 1.1-kb rRNA was greatly reduced (Fig 5B).

Consistent with a defect in processing of 23S rRNA which is a component of the chloroplast ribosome, the *rbd1* mutants had a reduction in chloroplast translated protein. When total leaf proteins were analyzed on a SDS polyacrylamide gel, the chloroplast Rubisco Large Subunit (RbcL) protein, which is synthesized in chloroplast, was found to have a drastic reduction in *rbd1-1* and *rbd1-2* mutants compared to the wild type by 4°C treatment but not at 22°C (Fig 5C). We further analyzed the chloroplast translation efficiency in the *rbd1* mutants. Spectinomycin is a chemical inhibitor of chloroplast translation as it prevents translocation of the peptidyl-tRNA from the A site to the P site on the 30S subunit of 70S ribosomes [39]. When seeds were sowed and grown on 1/2 MS supplied with spectinomycin at a concentration of 3mg/L at 22°C, both the *rbd1-1* and *rbd1-2* mutants exhibited total yellowing while the wild type and the complemented *rbd1* mutants maintained some green tissues (Fig 5D). This suggests that translation in chloroplast is compromised in the *rbd1* mutants even under non-chilling condition.

### The *rbd1* mutants exhibit a defect in 23S rRNA processing earlier than other defects

We further analyzed the transcripts of 9 chloroplast RNAs in the *rbd1* mutants under normal and chilling conditions to assess how broad a role the *RBD1* gene might play in chloroplast RNA regulation. They are 16S rRNA, PEP transcribed *ndhF*, *psaA*, *rbcL*, *psbB*, *psbF* and *petB*, NEP transcribed *ycf3*, and PEP and NEP transcribed *rps4*. Among these, 16S rRNA, *ndhF*, *psbB*, *petB*, *ycf3* and *rps4* need to be processed to become mature transcripts.

In contrast to that of 23S rRNA, the processing of 16S rRNA was only slightly affected in the *rbd1* mutants compare to Col-0 at 4°C but not 22°C (S3B Fig). The transcripts for *ndhF*, *psaA* and *rbcL* showed decreased levels under chilling stress compared to the wild-type plant, but displayed similar levels to wild type at 22°C (S3A Fig). For *psbB*, *psbF* and *petB*, low temperature down regulated their transcripts levels, but the transcripts were at similar levels in the *rbd1* mutants and wild type at 22°C and 4°C (S3B Fig). The transcripts of *ycf3-ex2* and *rps4* showed an over-accumulation under chilling condition in the *rbd1* mutants compared to the wild type, but exhibited the same levels at 22°C (S3C Fig). In all, the loss of *RBD1* mainly alters processing of 23S rRNAs but not other chloroplast RNAs we analyzed. It also reduces the expression level of some PEP transcribed genes, but increases the expression of some NEP transcribed genes at chilling temperature.

The leaf yellowing phenotype in the *rbd1* mutants likely results from an accumulative defect over time because it only became visible two weeks after plants were shifted from 22°C to 4°C. To identify earlier defects in the mutants, we monitored the molecular events after the plants were shifted from 22°C to 4°C at 0 hour (h), 6 h, 1 day (d), 3 d, 7 d, 14d, 21 d and 28 d. Among the 6 genes that showed altered processing and expression in the mutants, 23S rRNA was the first to show defects. Its processed 1.1-kb transcript was significantly lower than the wild type after 7 days of 4°C treatment (S4A Fig). The transcript of *psaA* showed defects in the *rbd1* mutants after 2 weeks of cold treatment (S4B Fig). The transcripts of the rest *ndhF*, *rbcL*, *ycf3-ex2* and *rps4* genes began to show significant change after 3 or 4 weeks of chilling treatment (S4 Fig). Therefore, the *rbd1* mutants exhibit a defect in 23S rRNA processing very early on (S4B, S4C and S4D Fig), which would lead to reduced chloroplast translation and bleaching phenotype under chilling conditions.

### RBD1 binds to 23S rRNA transcripts in a temperature dependent manner

**Fig 6:**
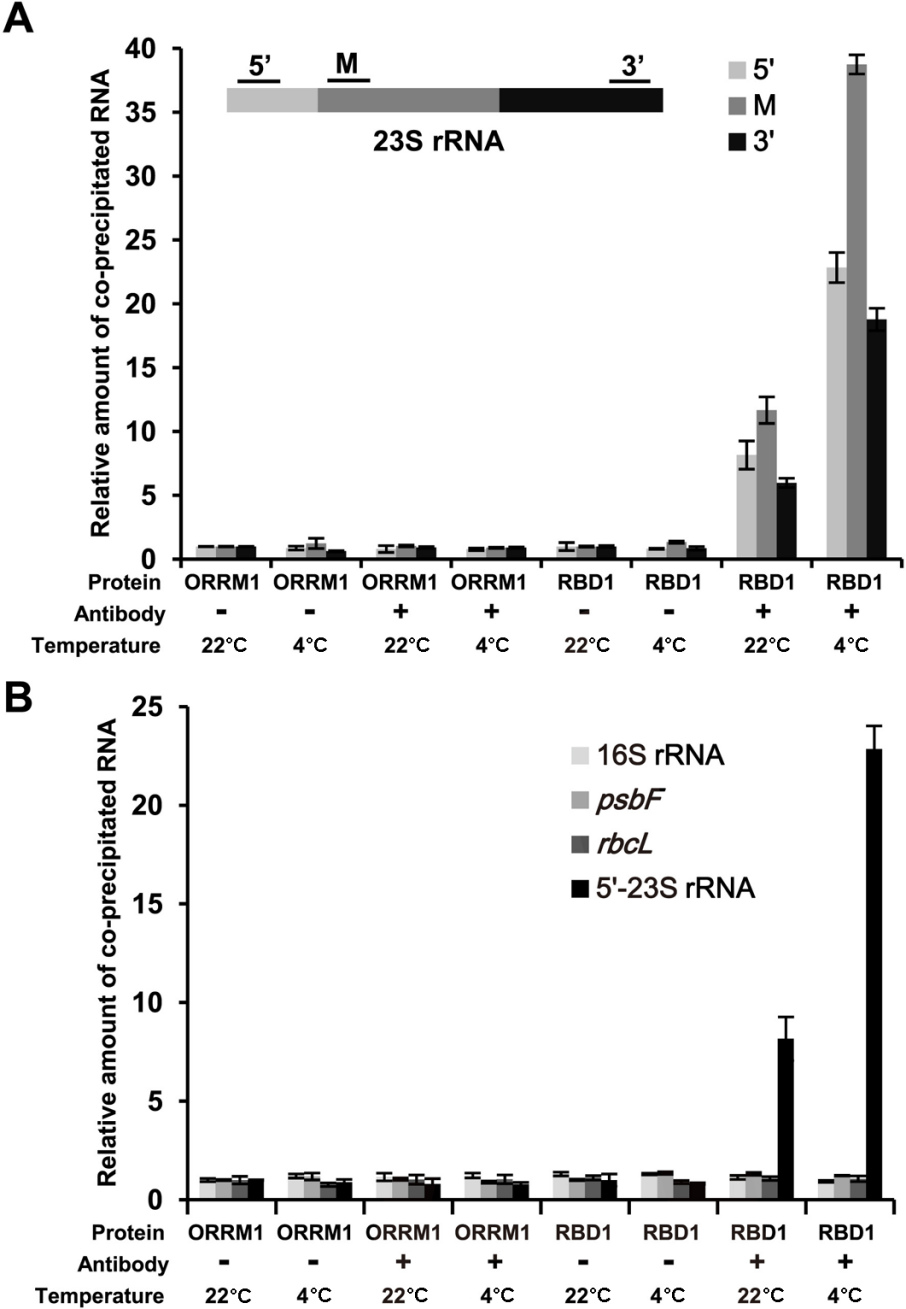
RBD1 binds to the 23S rRNA in a temperature dependent manner. (A) Shown are relative amounts of 23S rRNA transcripts co-immunoprecipitated (co-IPed) from RBD1 versus sORRM1 analyzed by qRT-PCR. Primer pairs for 5’-terminal (5’), middle part (M), and 3’-terminal (3’) of 23S rRNA were respectively used to amplify RNAs from immuno-precipitates after reverse transcription. DNase I was used to treat the precipitated samples before reverse transcription (RT), and samples not subject to RT were used as control in PCR to ensure there is no genomic DNA contamination. (B) Shown are relative amounts of transcripts of 16S rRNA, *psbF* and *rbcL* co-IPed from RBD1 versus sORRM1 analyzed by qRT-PCR. The GFP fusions of RBD1 (RBD1:GFP) and the signal peptide of ORRM1 (sORRM1:GFP) were expressed in protoplasts of wild-type Arabidopsis leaves at 22°C and 4°C, and anti-GFP antibodies were used to precipitate the GFP fusions. ‘-’ and ‘+’ mean no antibodies and added antibodies respectively.

To determine whether or not RBD1 protein is associated with the 23S rRNA and the association is temperature regulated, we performed RNA co-immunoprecipitation (IP) assay at both 22°C and 4°C. RBD1 was fused to the GFP (RBD1:GFP) and expressed under the strong 35S promoter in Arabidopsis protoplasts. As a control, the signal peptide of ORRM1 that targets the protein to chloroplast was fused with GFP (sORRM1:GFP) for protoplast expression as well. After transformation, the protoplasts were incubated at 22°C for 8 h before being split for further incubation at 22°C and 4°C respectively for 12 h to assess temperature dependency of binding. Total proteins from protoplasts were then IPed by the anti-GFP antibodies, and the co-IPed RNAs (after DNase I treatment) were reverse transcribed and detected by quantitative Real Time-PCR. We analyzed four transcripts: *23S rRNA*, *16S rRNA*, *psbF*, and *rbcL* and found binding only to *23S rRNA* (Fig 6). Three regions of the 23S rRNA precursor were analyzed: 5’ end, middle part, and 3’ end, residing in three different processed RNAs. There was an 8, 11 and 6 fold increase of PCR products for the three regions of 23S rRNA precursor in the RBD1:GFP sample compared to the sORRM:GFP sample at 22°C (Fig 6A). Furthermore, the fold increase for the three regions were 23, 39 and 19 fold in the RBD1:GFP sample compared to the sORRM:GFP at 4°C (Fig 6B). Therefore, RBD1 binds to three regions of 23S rRNA precursor, and the binding is at least two times more at 4°C than at 22°C. In contrast, no difference was detected for the 16S rRNA, *psbF* and *rbcL* between RBD1:GFP sand sORRM:GFP at either 22°C or 4°C (Fig 6A and 6B), suggesting a specificity of transcript binding for RBD1.

### Mutants of the 5 *RNP* genes have both specific and shared defects

**Fig 7:**
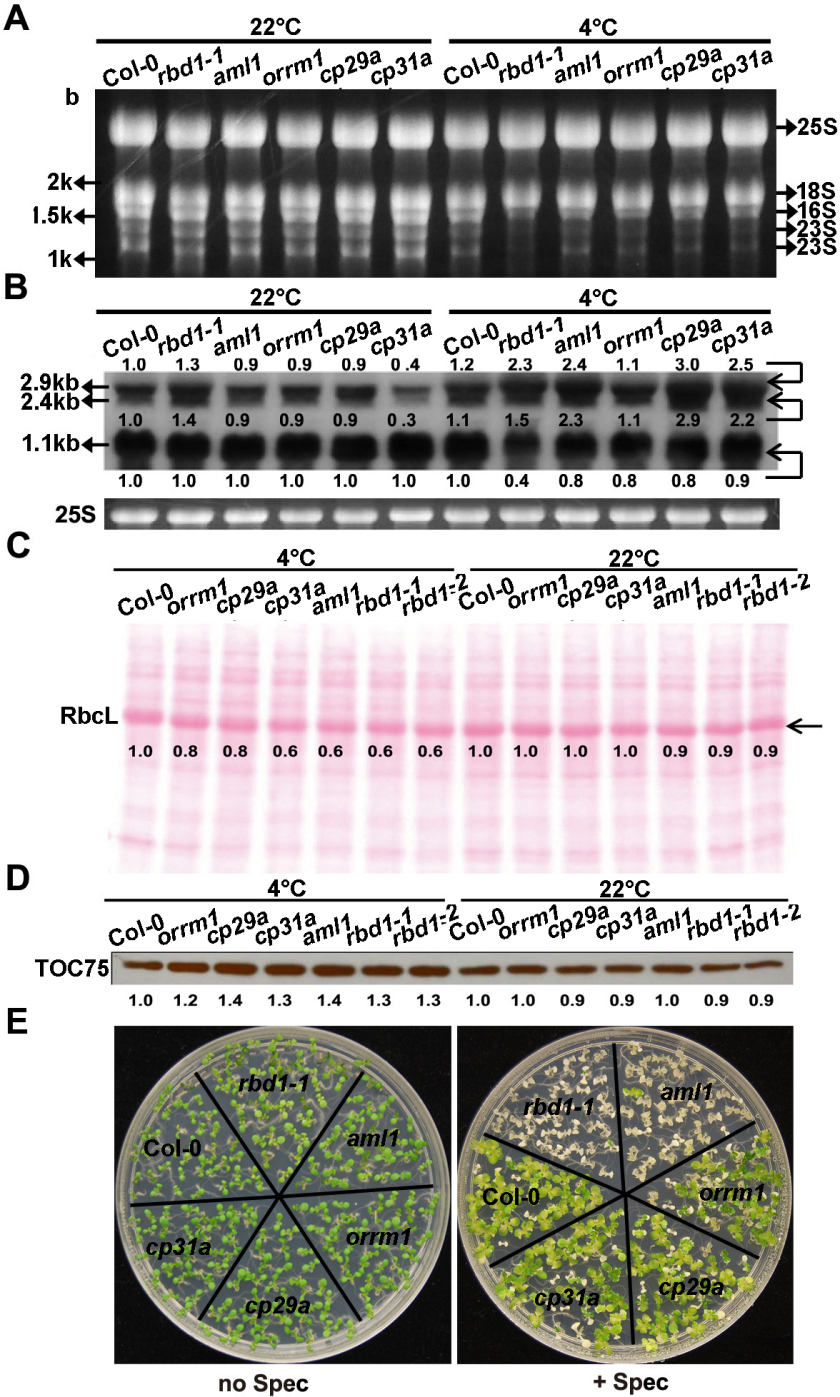
Analyses of chloroplast translation in the 5 *RNP* mutants. (A) Total RNAs extracted from the 5 *RNP* mutants were fractionated on 1.2% formaldehyde gels and stained with ethidium bromide. (B) RNA blotting analysis of 23S rRNA processing in the 5 *RNP* mutants. Total RNAs extracted from leaves were fractionated on 1.2% formaldehyde gels and hybridized to a probe described in Fig 5B. Equal loading was controlled by the cytosolic 25S rRNA (25S) stained with ethidium bromide. (C) Ponceau S Staining of total proteins from Col-0, *rbd1-1*, *aml1*, *orrm1*, *cp29a* and *cp31a* after SDS-PAGE separation. Accumulation of RbcL is indicated by an arrow. (D) TOC75 protein was detected by immune-blotting using specific monoclonal antibodies, loading control is shown in Fig 7C. (E) Shown are Col-0, *rbd1-1*, *aml1*, *orrm1*, *cp29a*, and *cp31a* mutants grown on solid media in the presence or absence of spectinomycin (spec) at 3 mg/L for 10 days. Plants were grown for 3 weeks at normal condition followed by 4°C for 4 weeks (A and B) or 2 weeks (C and D), and tissues were collected from five newly emerged leaves. Marked numbers indicate the relative amount in the mutant compared to that in Col-0 quantified by Image J.

We further compared the molecular defects among the 5 *RNP* mutants after 4 weeks of 4°C treatment. The *rbd1* mutants had the most drastic defect in 23S rRNA processing among the 5 *RNP* mutants. By RNA gel staining, reduction of processed 23S rRNAs compared to the wild type was the most pronounced in the *rbd1-1* mutant (Fig 7A). By RNA blotting, the processed 1.1-kb transcript was found to decrease in all of these 5 *RNP* mutants, with the most severe reduction happening in *rbd1-1*. The partially processed 2.9- and 2.4-kb transcripts were over-accumulated in these mutants at 4°C except for *orrm1* (Fig 7B). Among the 5 *RNP* mutants, the *rbd1-1* mutant exhibited the highest ratio of partially processed transcripts (2.4- and 2.9-kb) over processed transcripts (1.1-kb). Consistent with the observed 23S rRNAs processing defect in the 5 *RNP* mutants, the accumulation of RbcL protein at 4°C was reduced to different extent, with an approximately 20% to 40% reduction compared to the wild type (Fig 7C). In contrast to the RbcL protein, TOC75, a cytosol synthesize chloroplast outer membrane protein [40–42], showed an increased amount of 20% to 40% at 4°C in the 5 *RNP* mutants compared to the wild type (Fig 7D). In addition, *rbd1-1* showed the highest sensitivity to spectinomycin followed by *aml1* among the 5 *RNP* mutants assayed (Fig 7E). At 3mg/L concentration, all *rbd1-1* seedlings turned white and the *aml1* mutant was pale yellow, while the other three mutants stayed green similarly to the wild type. These results indicate that loss of *RBD1* has a larger effect on 23S rRNA processing compared to other mutants, which may impact chloroplast translation more than the others. It also shows that the *ORRM1* mutation has the least impact on rRNA processing.

## Discussion

In this genome-wide chilling sensitivity screen, we identified 54 T-DNA insertion lines that exhibited a chilling sensitive phenotype in Arabidopsis. Although we have not tested if all results from disruption of the gene that the T-DNA is inserted in, we expect that most of them are. Interestingly, a large proportion of these genes are related to chloroplast function, indicating that it is one of the weakest links in chilling tolerance. Earlier studies have implicated a strong association of compromised chloroplast function with chilling tolerance, and this study supports this notion at the genome wide scale. Wild-type Arabidopsis plants usually survive at low temperatures such as 4°C but mutants with compromised chloroplast function are sensitive to chilling similarly to the subtropical and tropical plants. To what extent difference in chilling tolerance among different plants is due to difference in chloroplast function at low temperatures is worth studying in the future. This screen also suggests that chilling tolerance and cold acclimation may not use identical mechanisms in Arabidopsis. Mutants of the known key regulators of the *CBF* pathway, which are unfortunately not present in the collection, are not known or reported to have chilling sensitive phenotypes. Mutants identified in the chilling sensitive screen here were not isolated as defective in the CBF pathways mutants. Indeed, the *rbd1-1* mutant is not defective in cold induction of the CBF pathway, suggesting that the induction of the *CBF* pathway may not require optimal chloroplast function and chilling sensitivity can result from *CBF* independent defect. Nevertheless, chilling tolerance can be connected with the CBF pathway. In Arabidopsis, a chilling sensitive mutant *crlk1* (*calmodulin receptor like kinase 1*) is chilling sensitive and is also delayed in *CBF* induction [43]. In rice, a chilling sensitive mutant *cold1* is defective in *CBF* induction [44]. It is possible that chilling sensitivity can result from defects in multiple processes and some might be shared with the *CBF* pathway.

The chloroplast localized proteins critical for chilling tolerance are involved in multiple aspects of chloroplast function, including transcript regulation and maturation, chloroplast development, protein transportation and secretion, as well as photosynthesis. For instance, *YS1* is required for editing of *rpoB* transcripts and chloroplast development during early growth [45]. *PQL3* is required for NDH activity in photosynthetic electron transport chain [46]. *ATTK1B* plays an important role in plant growth and development through the nucleotide salvage pathway [47]. Thus, the high proportion of chloroplast related genes in these mutants indicated a key role of chloroplast function in chilling tolerance in Arabidopsis.

We found in this study that RBD1, one of the chloroplast localized proteins identified in the chilling sensitive screen, is involved in 23S rRNA processing. The highly conserved *rrn* operon on chloroplast genomes encodes the 23S, 16S, 5S and 4.5S rRNAs and three tRNAs [48]. The primary precursor is initially processed to generate tRNAs, precursors of 23S, 16S, 5S and 4.5S rRNA by a series of endo- and exonucleolytic cleavages [49,50]. The partially processed 23S rRNA is then excised at two sites named the hidden breaks [38] (Fig 5B). Several molecules have been found to be involved in this final processing step. The CSP41b endonuclease and the RH39 RNA helicase are shown to be involved in processing the 23S rRNA at the 1st and 2nd hidden breaks respectively [36,38,51]. The RNase E endonuclease as well as the PNPase and RNase R exonucleases may also be involved in the final processing of 23S rRNA because their loss of function mutants accumulate incompletely processed 2.9-kb and 2.4-kb of the 23S rRNA, although their main function is to excise the 23S-4.5S species to generate 23S and 4.5S rRNAs [50,52,53]. Our study identifies RBD1 as a new regulator of 23S rRNA processing. Loss of *RBD1* causes over-accumulation of the partially processed 2.9-kb and the 2.4-kb of 23S rRNA and reduction of the fully processed 1.1-kb species while the total amount of 23S rRNA does not change significantly (Fig 5B). The RBD1 protein is localized to nucleoid where the processing happens (Fig 4C), and it binds to the 23S rRNA but not other RNAs tested (Fig 6). This indicates that RBD1 functions in the last step of 23S rRNA processing, and it may facilitate the processing by guiding the endonucleases to the hidden breaks. Further refining its RNA biding sequences and determining its interaction with processing enzymes will reveal more its mode of action. Compared to the other 4 *RNP* mutants with similar chilling sensitive phenotypes, the *rbd1-1* mutant exhibited the strongest 23S rRNA processing defect (Fig 7B). It has additional chloroplast RNA defects, but they did not occur at normal growth temperature and were induced at a much later stage after plants were chilling treated (S3 and S4 Figs). Because RBD1 does not appear to have enzymatic activities, it might interact with an RNase to bring it to the location for processing via its specific binding to the target RNA. Therefore, RBD1 is a positive facilitator of 23S rRNA processing. This function of RBD1 is more important at low temperatures than at normal temperatures. The *rbd1* mutants show a slight defect at normal growth temperature but very strong defect at 4°C (Fig 5B and S4A Fig). Interestingly, RBD1 exhibited a higher association with the 23S rRNA at 4°C than at 22°C, suggesting a regulation of its activity by temperature. The enhanced binding of RBD1 to the precursor could compensate for a less efficient binding of the processing enzyme RNase to 23S rRNA, so that chloroplast translation machinery can be efficiently produced at low temperatures as well.

It is still yet to be determined how chloroplast function, especially translation, is the weak link in chilling tolerance. Likely, RNAs in chloroplast form more non- or less- functional secondary structures under chilling temperatures. Such structures of rRNAs may compromise the assembly or function of the translation machinery, while those of mRNAs may reduce their translation efficiency. RNA binding proteins such as RBD1 and other RNPs may reduce the formation of such non- or less-functional structures and thus become more critical under chilling conditions.

Our study also indicates that various processes of RNA metabolism and protein translation in chloroplasts are inter connected. Different primary defects can have ripple effects leading to a similar chilling sensitive phenotype at later stages. For instance, RBD1 is closely associated with processing of the 23S rRNA transcripts. The primary defect of *rbd1* mutants is likely the reduced processing and accumulation of mature 23S rRNA products. This will lead to defect of plastid ribosome especially under chilling conditions and subsequently the translation defect in the chloroplasts. Since the core subunits of PEP are synthesized on plastid ribosomes, the *rbd1* mutants are expected to have reduced PEP level which will lead to a reduction in PEP-dependent mRNAs. As a compensation, the NEP dependent genes might become overexpressed [54]. Indeed, the chilling-treated *rbd1* mutants had reduced transcript levels of the PEP-dependent genes tested such as *ndhF*, *psaA* and *rbcL*, while NEP-dependent genes such as *ycf3* had higher expression. *rps4*, transcribed by both PEP (in green tissue) and NEP (in white tissue) [55], had an overexpression in *rbd1* mutants under chilling condition (S3 Fig). The slight deficiency in 16S rRNA processing might be an indirect effect of losing the RBD1 function because abnormal ribosome assembly have been shown to indirectly affect RNA processing [56,57]. Interestingly, the *rbd1* mutants had a reduced level of chloroplast synthesized RbcL protein, but increased level of cytosol translated TOC75 protein (Fig 7D), suggesting a compensation at the translation level as well.

Together, this study reveals the importance of chloroplast RNA-binding proteins in chilling tolerance, and further studies will further enhance our understanding of molecular mechanisms of chilling tolerance and its variations in different plant species.

## Materials and Methods

### Plant materials and growth conditions

Arabidopsis T-DNA insertion lines were obtained from Arabidopsis Biological Resource Center with the stock numbers CS27941, CS27942 and CS27943. Four seeds of each line were sterilized and sowed on 0.5× MS (Murashige and Skoog, Sigma) solid medium with 1.2% agar and 2% sucrose. Plants were grown on vertical plates under a 16 h-light/d photoperiod for 8 days before being transferred to 4°C for two months. When using soil, plants were grown under a 12h-light photoperiod with light intensity at 100 μmol m^-2^ sec^-1^ and relative humidity at 50–70%. For spectinomycin treatment, seeds were sterilized and planted on 0.5×MS medium containing 0.8% agar and 2% sucrose with 0–3 mg/L spectinomycin under the conditions described above.

### Plasmid construction

For complementing the *rbd1* mutants, a full-length At1g70200 cDNA was amplified and cloned into PMDC32 [58] to generate the construct of p35S::RBD1. For the promoter reporter construct pRBD1::GUS, a 1.5-kb sequence upstream of the RBD1 translation start site was amplified and cloned into pCAMBIA1300 vector (CAMBIA, http://www.cambia.org). To generate a GFP-tagged RBD1 fusion protein (RBD1:GFP) for transient expression in protoplasts, the coding region of the RBD1 was amplified from the cDNA and cloned into the pSAT6-EGFP-N1 vector [59].

### Generation of transgenic plants

*Agrobacterium tumefaciens* stains of GV3101 carrying the resulting constructs were used to transform plants by standard floral dipping [60]. Primary transformants were selected on 0.5×MS (Murashige and Skoog, Sigma) medium containing 0.8% agar and 2% sucrose with 25 mg/L hygromycin for selection.

### Protoplast transformation

Protoplast isolation and transformation were carried out as previously described [61]. In brief, protoplasts were generated from 14-day-old wild-type Arabidopsis seedlings grown on plates with 12h light/ 12h darkness photoperiod and transformed with the plasmid DNAs. Protoplasts were then analyzed for GFP signals or for RNA co-IP from 12 hours to 48 hours after transformation.

### Histochemical GUS staining

GUS staining was performed as described previously [62]. Briefly, tissues were stained with X-Gluc staining buffer (5 mM potassium ferrocyanide, 5 mM potassium ferricyanide,100 mM sodium phosphate buffer, pH 7.0, and 0.005% Triton X-100 and 2 mM 5-bromo-4-chloro-3-indolyl-beta-D-glucuronic acid) for 1–2 hours at 37°C, followed by incubating in 70% ethanol to remove chlorophyll.

### Real-time quantitative PCR and gene expression

Total RNA was extracted with TRIzol reagent (Invitrogen) according to the manufacturer’s protocol. cDNAs were synthesized from total RNA by using AffinityScript QPCR cDNA Synthesis Kit (Agilent Technologies). Real-time quantitative PCR was performed on the BIO-RAD PCR System using iQSYBR GREEN SuperMix (BIO-RAD). *Actin* was used as a control.

### RNA gel blot analyses

Total RNA was extracted with TRIzol reagent (Invitrogen) according to the manufacturer’s instructions. For transcript analysis, five youngest visible leaves were harvested for RNA extraction. Ten micrograms of RNA per sample were separated on an agarose gel containing 1.2% formaldehyde and then transferred to uncharged nylon membranes (HybondN; GE Healthcare). The blots were UV cross-linked (150 mJ/cm^2^) and hybridized with gene specific, ^32^P labeled, single strand DNA probes.

### Protein analysis

Arabidopsis leaves were quick frozen in liquid nitrogen and homogenized in extraction buffer (50mM Tris-HCl, 1mM EDTA, 1mM EGTA, 150mM NaCl, 10% Glycerol, 5mM DTT, 0.25% Triton-X 100, 2% PVPP, 1mM PMSF). After centrifugation at 14,000 rpm for 10 min twice, the supernatants were mixed with loading buffer, boiled and loaded onto 12% SDS polyacrylamide gel. The proteins were visualized with Coomassie Blue staining of the gel or Ponceau S staining of the transferred blot.

### RNA co-immunoprecipitation

After transformation, the protoplasts were incubated at 22°C for 8 h before half of each sample was transferred to 4°C and the other half to 22°C for 12 h. Protoplasts were then disrupted in 500 μL protoplast disruption buffer (0.3M sorbitol, 20mM Tricine-KOH (pH 8.4), 10 mM EDTA, 10 mM NaHCO_3_ and 0.1% BSA) and incubated on the ice for 30 minutes with several inversion, then were centrifuged at 300×g for 2 minutes. Poured off the supernatant, chloroplasts pellet were disrupted in 200 μL chloroplast disruption buffer (2mM DTT, 200 mM KOAC, 30 mM HEPES, pH 8.0, 10 mM MgOAc, and 2mg/ml proteinase inhibitor cocktail) and incubated on the ice for 30 minutes with occasional roughly vortex, then were centrifuged at 16,000×g for 30 minutes at 4°C. The supernatant was diluted with one volume of Co-IP buffer (150 mM NaCl, 20 mM Tris-HCl, 1 mM EDTA, 5 mM MgCl_2_, 1.1% Triton X-100, 100U/ml RNase inhibitor, 1 mM PMSF and 2mg/ml proteinase inhibitor cocktail) and incubated with 10 μg of GFP antibody (mouse IgG2a, Invitrogen) for 6 h with rotation, followed by 2 h rotation with 50 μL Dynabeads Protein G (Invitrogen) at 4°C. Beads containing the IPed protein and its bound RNAs were collected with a magnet, and supernatants were recovered and pellets were washed three times with Co-IP buffer. After the last wash, pellets were resuspended in 200μL Co-IP buffer. Resuspension was then extracted with TRIzol reagent (Invitrogen) according to the manufacturer’s protocol. Total RNA was treated by DNase I (Promega) to remove DNA contamination, and RT-minus control was performed to confirm complete removal of DNA in the following steps. cDNAs were synthesized from total RNA by using Superscript III reverse transcriptase (Invitrogen). Real-time quantitative PCR was performed on the BIO-RAD PCR System using iQSYBR GREEN SuperMix (BIO-RAD).

## Supporting Information

S1 Fig Flow chart of screening chilling sensitive mutants. (TIF)

S2 Fig Analysis of RBD1 localization at 22°C and 4°C. Shown are confocal microscope images of protoplasts expressing RBD1:GFP at 22°C and 4°C. Left panels show the GFP signals, middle panels show the chlorophyll signals, and the right panels show the merged signals.
(TIF)

S3 Fig Analysis of accumulation and processing of chloroplast transcripts in *rbd1* mutants at 22°C and 4°C. Shown are transcripts with reduced (A), similar (B) and increased (C) expression level after chilling treatment relative to wild-type controls. Plants were grown for 3 weeks at normal condition followed by 4°C for 4 weeks, and tissues were collected from five newly emerged leaves. Marked numbers indicate the relative amount in the mutant compared to that in Col-0 quantified by Image J.
(TIF)

S4 Fig Time-course analyses of chloroplast transcripts accumulation and processing in *rbd1* mutants at chilling condition. (A) Shown are transcripts with processing defects after chilling treatment compared to the wild type. (B-C) Shown are transcripts with reduced (B) and increased (C) expression level after chilling treatment relative to wild-type controls. (D) Schematic diagram of the chloroplast transcript defects at different chilling treatment time points. For A, B, and C, plants were grown for 3 weeks at normal condition followed by 4°C for 4 weeks, and tissues were collected from five newly emerged leaves.
(TIF)

S1 Table Primers used in this study. (DOC)
