# Supplementary figures and images for: Chloroplast RNA-Binding Protein RBD1 Promotes Chilling Tolerance through 23S rRNA Processing in Arabidopsis
Source: PLoS Genet. 2016 May 3;12(5):e1006027. doi: 10.1371/journal.pgen.1006027 (PMC4854396; doi:10.1371/journal.pgen.1006027)

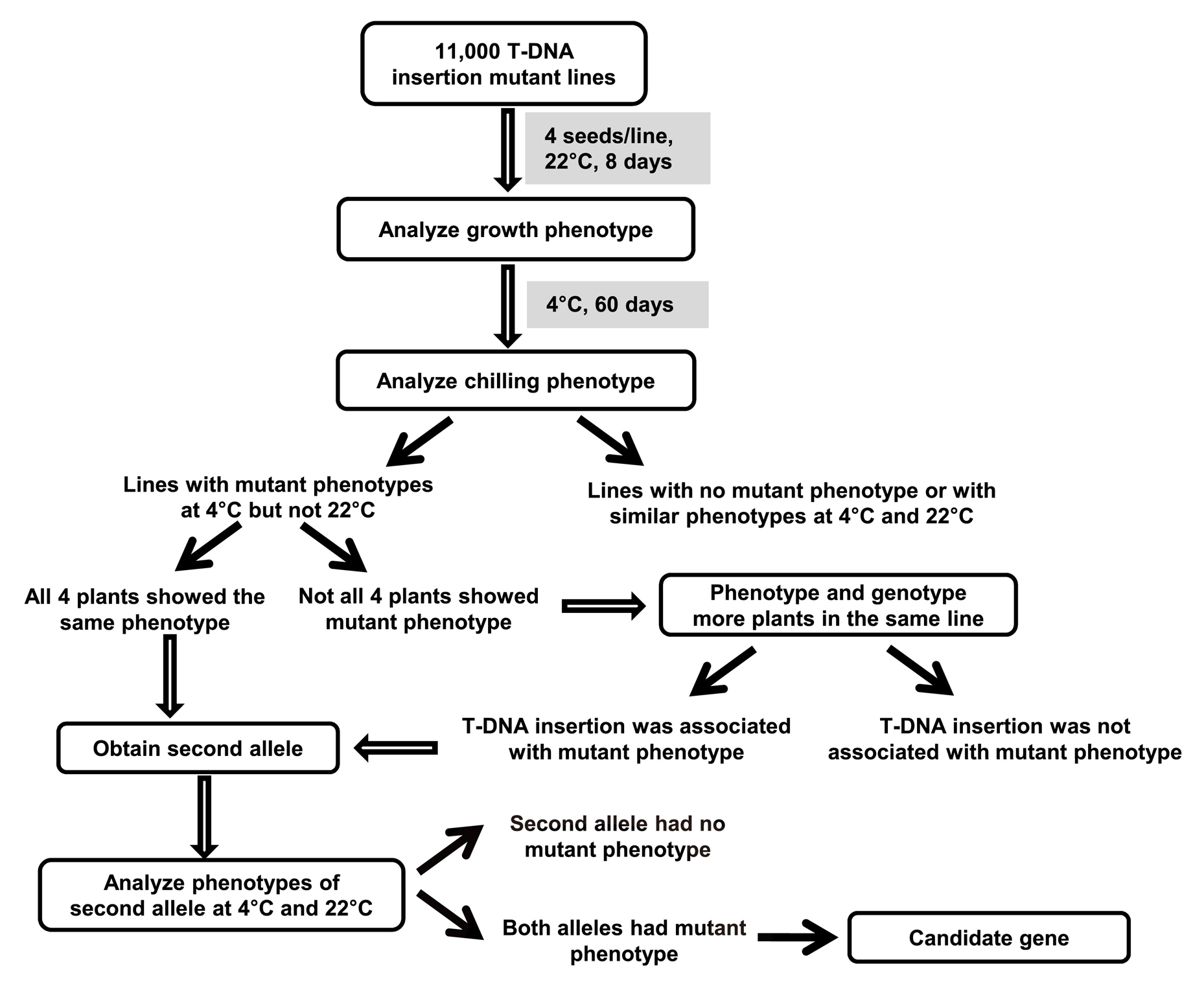

Supplement: S1 Fig — (TIF) [file pgen.1006027.s001.tif]

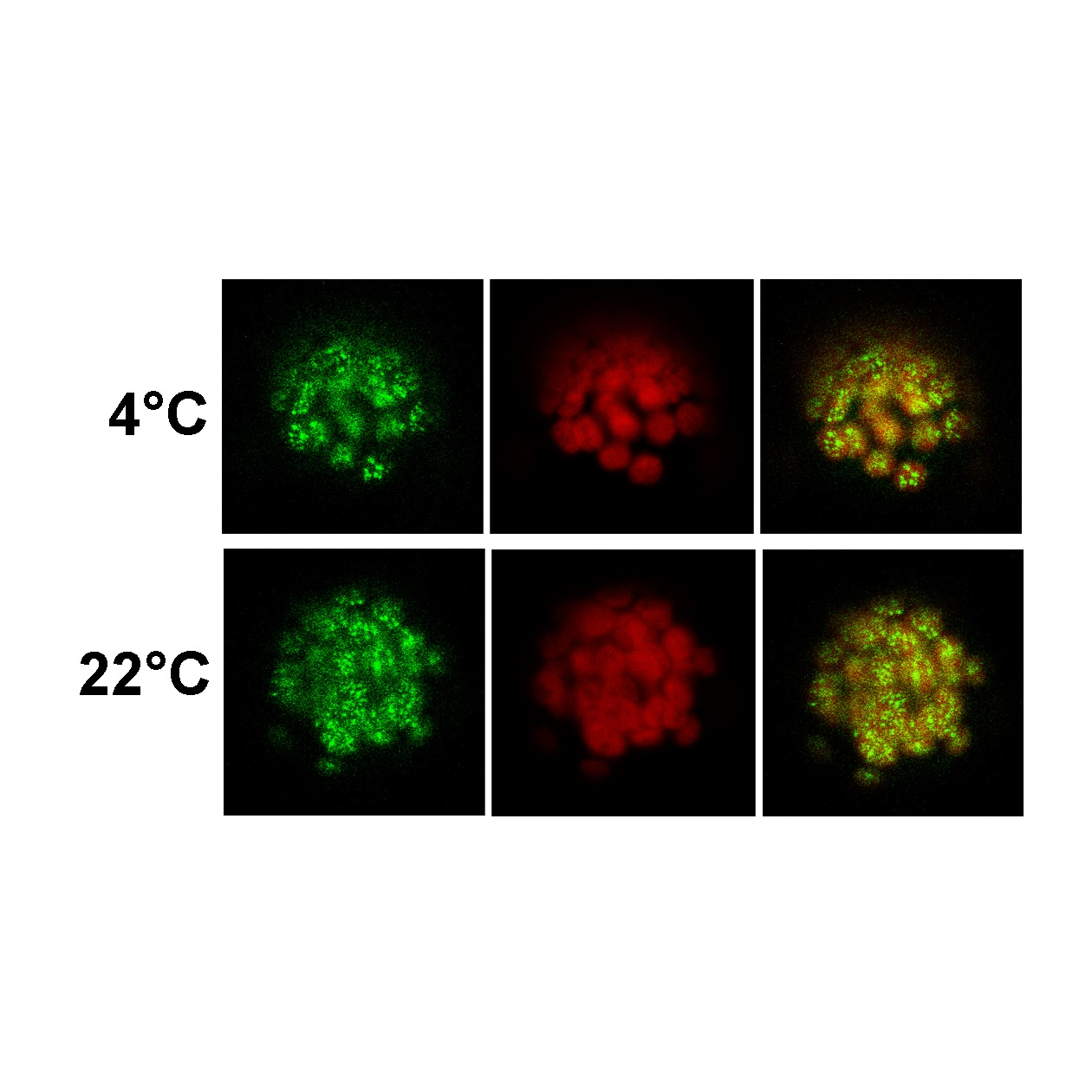

Supplement: S2 Fig — Shown are confocal microscope images of protoplasts expressing RBD1:GFP at 22°C and 4°C. Left panels show the GFP signals, middle panels show the chlorophyll signals, and the right panels show the merged signals. (TIF) [file pgen.1006027.s002.tif]

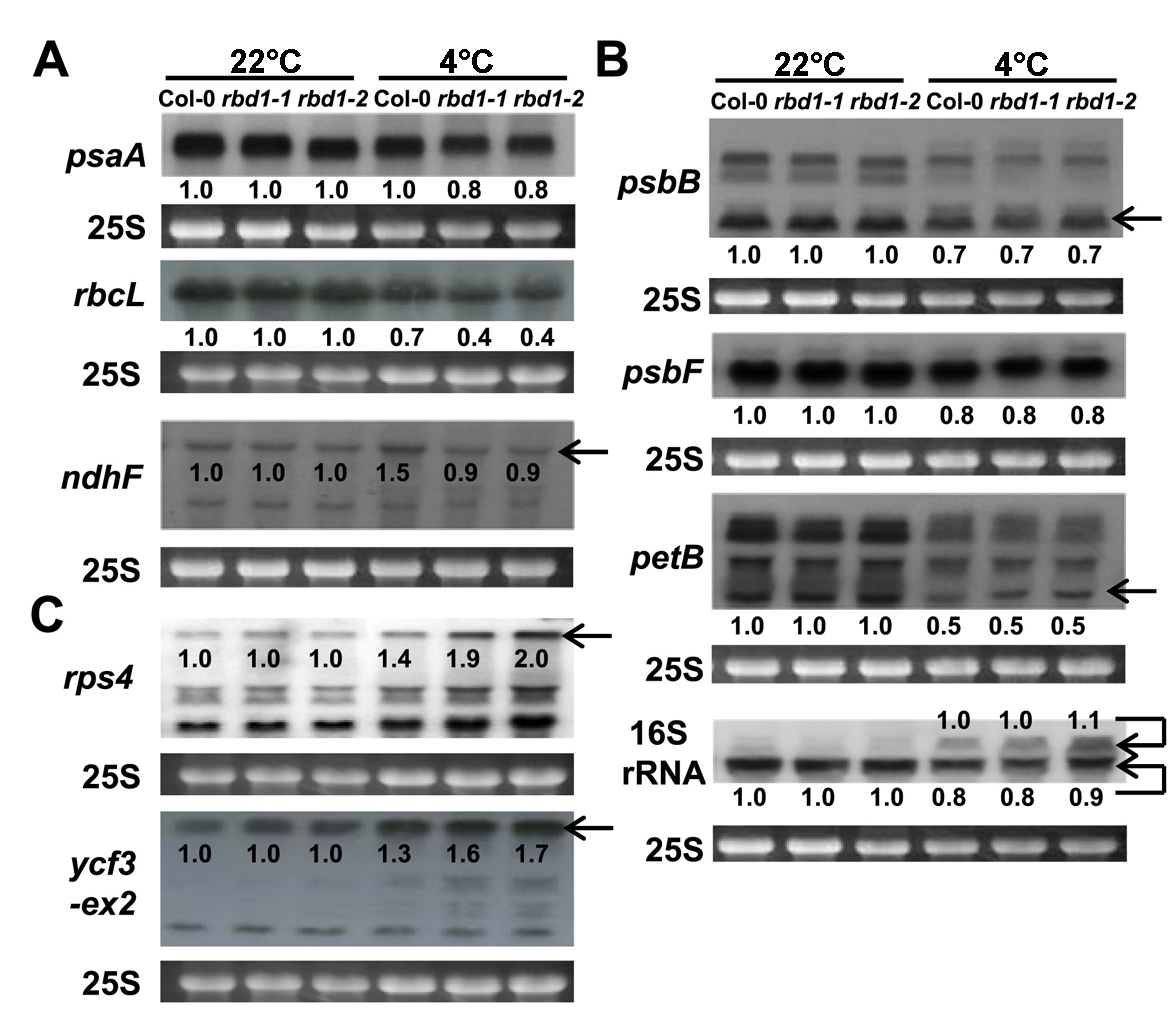

Supplement: S3 Fig — Shown are transcripts with reduced (A), similar (B) and increased (C) expression level after chilling treatment relative to wild-type controls. Plants were grown for 3 weeks at normal condition followed by 4°C for 4 weeks, and tissues were collected from five newly emerged leaves. Marked numbers indicate the relative amount in the mutant compared to that in Col-0 quantified by Image J. (TIF) [file pgen.1006027.s003.tif]

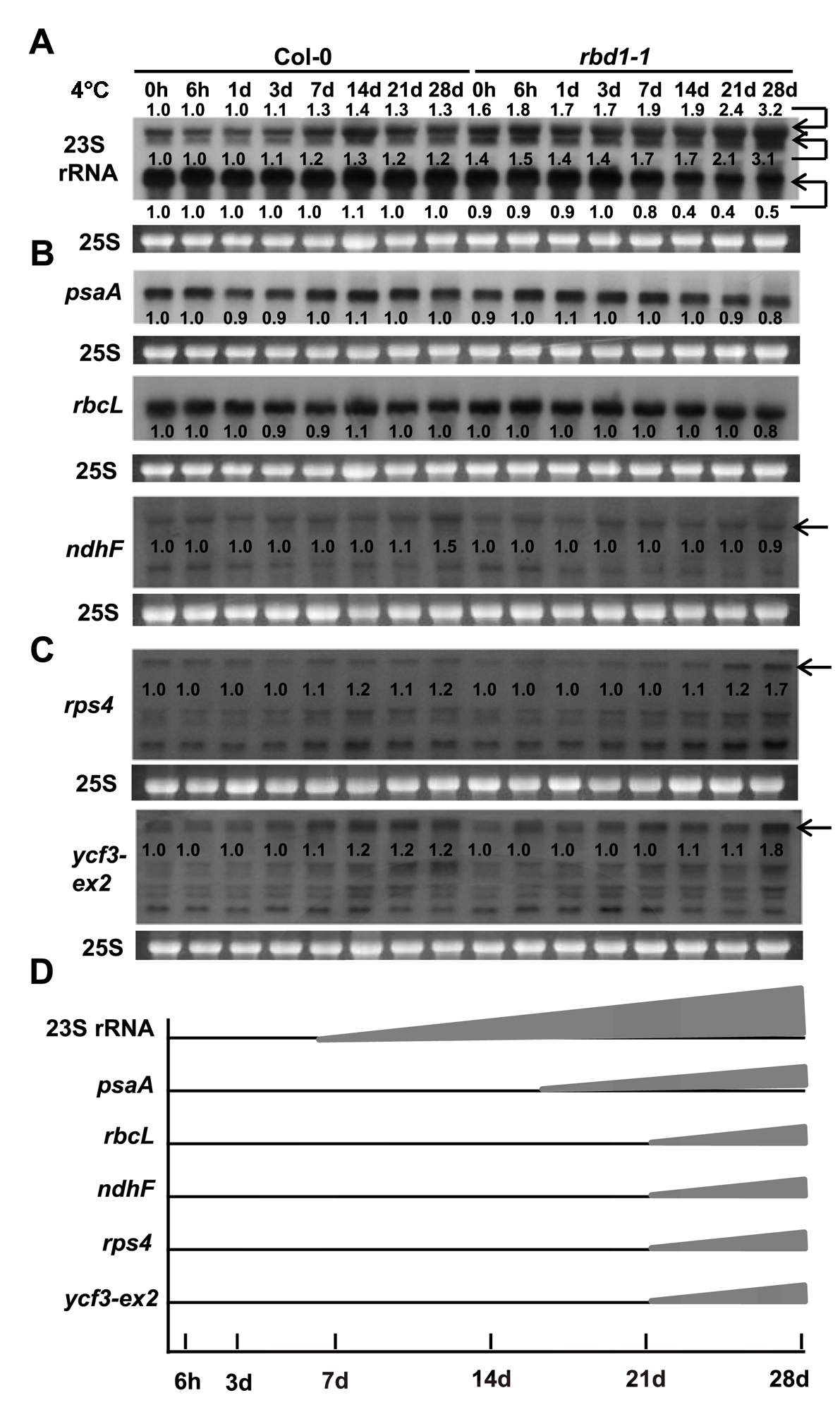

Supplement: S4 Fig — (A) Shown are transcripts with processing defects after chilling treatment compared to the wild type. (B-C) Shown are transcripts with reduced (B) and increased (C) expression level after chilling treatment relative to wild-type controls. (D) Schematic diagram of the chloroplast transcript defects at different chilling treatment time points. For A, B, and C, plants were grown for 3 weeks at normal condition followed by 4°C for 4 weeks, and tissues were collected from five newly emerged leaves. (TIF) [file pgen.1006027.s004.tif]
