# Supplementary material for: Chloroplast RNA-Binding Protein RBD1 Promotes Chilling Tolerance through 23S rRNA Processing in Arabidopsis
Source: PLoS Genet. 2016 May 3;12(5):e1006027. doi: 10.1371/journal.pgen.1006027 (PMC4854396; doi:10.1371/journal.pgen.1006027)
Supplement: S1 Table — (DOC) [file pgen.1006027.s005.doc]

**S1 Table. Primers used in this study.**

| Name | Sequence (5'>3') | Target(s) | Purpose |
| --- | --- | --- | --- |
| SALK_041100-LP | GTGAAACAGTTCACGGGACAGA | *RBD1* | Genotyping |
| SALK_041100-RP | CTTCCTCAGATTTATTCACCTGG | *RBD1* | Genotyping |
| SALK_012657-LP | GTACACAGTACACTCAAAAGCT | *RBD1* | Genotyping |
| SALK_012657-RP | CTTTGAGGAGATTATCGTCAC | *RBD1* | Genotyping |
| LB | ATGGTTCACGTAGTGGGCCATCG | T-DNA | Genotyping |
| AT1G70200-F | ATGAACGGAGCTTCGCTCTGC | *RBD1* | Gene cloning |
| AT1G70200-R | AGCTATGTGTTTTCTTTTCGTTATC | *RBD1* | Gene cloning and qRT-PCR |
| AT1G70200-R2 | TTCATCATTGTTTGAGGTTGAAGC | *RBD1* | Gene cloning (no stop codon) |
| AT1G70200-RNAi-F | CGGGATCCATTTAAATTGGGTTTTGTTCATTCGCTGTAAA | *RBD1* | RBD1-RNAi |
| AT1G70200-RNAi-R | GACTAGTCGGCGCGCCTTCTTCTTCTTAGGAAGATTAGTC | *RBD1* | RBD1-RNAi |
| AT1G70200promoter-F | CTGCAGGTACACAGTACACTCAAAAGCT | *RBD1* | RBD1 promoter cloning |
| AT1G70200promoter-R | CCCGGGAGCTATGTGTTTTCTTTTCGTTATC | *RBD1* | RBD1 promoter cloning |
| AT1G70200RT-F | TGGCTTTGATAAACCAGAAGC | *RBD1* | qRT-PCR |
| Actin-F | GGATCTGTACGGTAACATTG | *ACTIN* | Internal qRT-PCR control |
| Actin -R | AACGATTCCTGGACCTGCC | *ACTIN* | Internal qRT-PCR control |
| psbB-F | TTTGCAGCTTTTGTTGTTGC | *psbB* | RNA blotting |
| psbB-R | CTTCTAAACGGGACGTCAGC | *psbB* | RNA blotting |
| petB-F | CGTCCAACCGTTACTGAAGC | *petB* | RNA blotting |
| petB-R | AATAGCGTCAGGTACACC | *petB* | RNA blotting |
| psaA-F | TGGCATGTATTTCCATGGTG | *psaA* | RNA blotting |
| psaA-R | AACCAAGCCAATTTTGGAG | *psaA* | RNA blotting |
| psbF-F | GTCTGGAAGCACAGGAGAACG | *psbF* | RNA blotting and qRT-PCR |
| psbF-R | CAAAACGGCCTGTTATTAATGG | *psbF* | RNA blotting and qRT-PCR |
| rbcL-F | GCAGCATTCCGAGTAACTCC | *rbcL* | RNA blotting and qRT-PCR |
| rbcL-R | CCACGTAGACATTCATAAACTGC | *rbcL* | RNA blotting and qRT-PCR |
| rrn16-F | ATGGATACTAGGCGCTGTGC | 16S rRNA | RNA blotting and qRT-PCR |
| rrn16-R | ACCTTCCTCCGGCTTATCAC | 16S rRNA | RNA blotting and qRT-PCR |
| ycf3_exon2-F | CGGATGTCGGCTCAATCTGAAGG | *ycf3* | RNA blotting |
| ycf3_exon2-R | AGAGGGGTTTCGTTCTAATGCCCGA | *ycf3* | RNA blotting |
| ndhF-F | TTTTTCACGCCGTCAATAAACC | *ndhF* | RNA blotting |
| ndhF-R | AGAAGAGATGCGACTTCCAC | *ndhF* | RNA blotting |
| rrn23-3'-F | GCAAGACCCACCCGTCGAGC | 23S rRNA | RNA blotting |
| rrn23-3'-R | CGCTCCGCACTTGGCTACCC | 23S rRNA | RNA blotting |
| rps4-F | CGTCTGGGGGCTTTACCGGG | *rps4* | RNA blotting |
| rps4-F | TGGTTTGGCAATTCCTCAGGGGC | *rps4* | RNA blotting |
| 23S rRNA5RIP-F | ACGAGGAAGGGCGTAGTAAG | 23S rRNA | qRT-PCR |
| 23S rRNA5RIP-R | TCAGCAGCAGTTCAAAAGGT | 23S rRNA | qRT-PCR |
| 23S rRNAMRIP-F | CAGTGAGACGGTGGGGGATA | 23S rRNA | qRT-PCR |
| 23S rRNAMRIP-R | AGGGTGGCTGCTTCTAGGCA | 23S rRNA | qRT-PCR |
| 23S rRNA3RIP-F | ATATCCGGTGTGGGCGTTAG | 23S rRNA | qRT-PCR |
| 23S rRNA3RIP-R | GTTATCCGCTCCGCACTTGG | 23S rRNA | qRT-PCR |
| CBF1-F | GTGACGTGTCGCTTTGGAGTTAC | *CBF1* | qRT-PCR |
| CBF1-R | GTGAAGCAAAGAAGTAGAAAACG | *CBF1* | qRT-PCR |
| CBF2-F | TCGAGGGAGATGATGACGTGTCC | *CBF2* | qRT-PCR |
| CBF2-R | TATTTTGATTTGTTGCTTATGG | *CBF2* | qRT-PCR |
| CBF3-F | CGACGGCGATGATGACGACGT | *CBF3* | qRT-PCR |
| CBF3-R | GCATTTAAGAATAGCCCACAC | *CBF3* | qRT-PCR |
| COR15A-F | ATGTCTTTCTCAGGAGCTGTTC | *COR15A* | RNA blotting |
| COR15A-R | CCTACTTTGTGGCATCCTTAGC | *COR15A* | RNA blotting |
| COR47-F | ATGGCTGAGGAGTACAAGAAC | *COR47* | RNA blotting |
| COR47-R | ACTTCCTCTTCAGTGGTCTTGG | *COR47* | RNA blotting |
| KIN1-F | ATGTCAGAGACCAACAAGAATG | *KIN1* | RNA blotting |
| KIN1-R | CCGAATCTCTACTTGTTCAGG | *KIN1* | RNA blotting |

Underline indicates added sequences to the gene sequences for cloning purpose.
